# Time-Course RNA-Seq Analysis Reveals Transcriptional Changes in Rice Plants Triggered by *Rice stripe virus* Infection

**DOI:** 10.1371/journal.pone.0136736

**Published:** 2015-08-25

**Authors:** Won Kyong Cho, Sen Lian, Sang-Min Kim, Bo Yoon Seo, Jin Kyo Jung, Kook-Hyung Kim

**Affiliations:** 1 Department of Agricultural Biotechnology and Plant Genomics and Breeding Institute, College of Agriculture and Life Sciences, Seoul National University, Seoul, 151–921, Republic of Korea; 2 Crop Protection Division, National Academy of Agricultural Science, RDA, Suwon, 441–707, Republic of Korea; 3 Crop Environment Research Division, National Institute of Crop Science, RDA, Suwon, 441–857, Republic of Korea; 4 Research Institute of Agriculture and Life Sciences, Seoul National University, Seoul, 151–921, Republic of Korea; National University of Singapore, SINGAPORE

## Abstract

*Rice stripe virus* (RSV) has become a major pathogen of rice. To determine how the rice transcriptome is modified in response to RSV infection, we used RNA-Seq to perform a genome-wide gene expression analysis of a susceptible rice cultivar. The transcriptomes of RSV-infected samples were compared to those of mock-treated samples at 3, 7, and 15 days post-infection (dpi). From 8 to 11% of the genes were differentially expressed (>2-fold difference in expression) in RSV-infected vs. noninfected rice. Among them, 532 genes were differentially expressed at all three time points. Surprisingly, 37.6% of the 532 genes are related to transposons. Gene ontology enrichment analysis revealed that many chloroplast genes were down-regulated in infected plants at 3 and 15 dpi. Expression of genes associated with cell differentiation and flowering was significantly down-regulated in infected plants at 15 dpi. In contrast, most of the up-regulated genes in infected plants concern the cell wall, plasma membrane, and vacuole and are known to function in various metabolic pathways and stress responses. In addition, transcripts of diverse transcription factors gradually accumulated in infected plants with increasing infection time. We also confirmed that the expression of gene subsets (including NBS-LRR domain-containing genes, receptor-like kinase genes, and genes involving RNA silencing) was changed by RSV infection. Taken together, we demonstrated that down-regulation of genes related to photosynthesis and flowering was strongly associated with disease symptoms caused by RSV and that up-regulation of genes involved in metabolic pathways, stress responses, and transcription was related to host defense mechanisms.

## Introduction

*Rice stripe virus* (RSV) belongs to the genus *Tenuivirus* but has not been assigned to a virus family [1]. RSV is transmitted by the small brown planthopper (SBPH; *Laodelphax striatellus*) in a persistent circulative-propagative manner [1]. The host of RSV, rice (*Oryza sativa* L.), is an important cereal crop worldwide and in scientific research is a model plant for monocot species [2]. During the last few decades, RSV has become a major pathogen of rice in Asian countries including Korea, China, and Japan [1, 3, 4]. The genome of RSV is composed of four RNAs that encode seven proteins with an ambisense coding strategy [1].

To prevent yield losses caused by RSV, researchers have attempted to find RSV-resistant cultivars [5]. For example, the resistance gene *STV*-*b* ^i^, which was bred from the *indica* paddy rice Modan into *japonica* rice by backcrossing, has been used to control RSV in Japan and Korea for over 40 years [6]. In another approach to minimize RSV damage, researchers have developed several transgenic rice plants [7].

To understand the mechanism of RSV infection and RSV resistance in rice and to identify important genes involved in the rice–RSV interaction, large-scale analyses such as transcriptomic- based approaches can be useful. Thus, microarray-based transcriptomic approaches have been used to identify genes related to RSV infection or symptom development [8]. Furthermore, with the rapid development of next generation sequencing, comparative transcriptome analysis of two rice varieties in response to RSV infection has been conducted [9]. In addition, rice miRNAs associated with RSV infection as well as small interfering RNAs from RSV have been identified by small RNA deep sequencing [10, 11].

RSV infection causes various symptoms including yellow strips and necrotic streaks on leaves and the failure of emerging leaves to unfold properly such that they droop. Early infection by RSV causes severe stunting, malformation of emerging leaves, and even death, resulting in severe yield losses [12]. RSV infection at later growth stages reduces tiller number, retards heading, and degeneration of spikelets resulting in infertility [13]. The level of yield loss of a susceptible rice cultivar such as Nipponbare is very closely related to plant stage at time of infection, i.e., yield loss is much greater when young plants rather than more mature plants are infected.

In the present study, we used RNA-Seq to conduct a time-course analysis of the rice transcriptome in response to RSV infection. A total of six libraries including RSV-infected and mock-treated samples from three time points were subjected to RNA-Seq analysis. Our study provides a genome-wide gene expression profiles in response to RSV infection on a susceptible rice cultivar.

## Materials and Methods

### Plant Material

To monitor the response of the rice transcriptome to RSV infection, we selected the RSV-susceptible rice cultivar Nipponbare (*Oryza sativa* L. ssp. *Japonica*) for which the full genome sequence and gene annotation are freely available [2]. The rice seeds were kindly provided by the RDA-Genebank Information Center (http://www.genebank.go.kr/).

### Rice Seedlings and RSV Infection

Rice seeds were planted in a commercial rice nursery medium (Punong Co. LTD, Kyoungju, Korea) and were grown in a greenhouse at the National Institute of Crop Science, Miryang, Korea. Based on a previously reported method [14], 2-week-old seedlings (typically at the two- to three-leaf stage) were infested with viruliferous SBPH containing RSV or with non-viruliferous SBPH for RSV-infected and mock-treated samples, respectively. Each seedling was exposed to four to five 2^nd^ and 3^rd^ instar nymphs of SBPH. After 2 days, all SBPHs were removed from the seedlings.

### Samplings of Seedlings and Total RNA Extraction

RSV-infected and mock-treated samples were collected at 3, 7, and 15 days post-infection (dpi), i.e., 3, 7, and 15 days after exposure to SBPH. Five seedlings for each condition were harvested. At each time point, 1 to 2 cm of a leaf blade was cut from each seedling and immediately frozen in liquid nitrogen. Total RNA was extracted using the Qiagen RNeasy plant mini kit (Qiagen GmbH, Hilden, Germany) following the manufacturer’s instructions. Total RNA prepared from each sample was used for RT-PCR to confirm that samples were or were not RSV-infected at each time point. First-strand cDNA was synthesized using GoScript Reverse Transcriptase (Promega Corp, Madison, U.S.A.), and PCR amplification was done using Takara Ex Taq Polymerase (Takara, Kusatsu, Japan) and specific primers that amplified partial sequences of the RSV nucleocapsid gene (forward primer: CTAGTCATCTGCACCTTCTG and reverse primer: ACTTACTGTGGGACTATGTT) following manufacturer’s instructions. Total RNA samples of confirmed RSV-infected seedlings and mock controls were used for library construction.

### Preparation of Libraries for RNA-Seq

The quality of total RNA was checked with an Agilent 2100 bioanalyzer (Agilent Technologies, Santa Rosa, USA), and RNA samples with RNA integrity numbers > 7 were selected for library preparation. To reduce variation among samples for each time point, the same quantity of total RNAs from three samples were mixed together. A total of six multiplexed libraries for next generation sequencing were prepared using the TruSeq RNA Sample Prep Kit v2 (Illumina, San Diego, U.S.A.) according to the manufacturer’s instructions. Paired-end sequencing was performed by Illumina HiSeq 2000 at the National Instrumentation Center for Environmental Management (NICEM, Seoul, Korea).

### Mapping, Normalization, and Calculation of RPKM

The obtained raw data (12 fastq files) were deposited in the National Agricultural Biotechnology Information Center (NABIC) (http://nabic.rda.go.kr) with the following accession numbers (NN-0735, NN-0736, NN-0745, NN-0747, NN-0872 to NN-0879). Release 7 of the rice reference sequence was downloaded from the rice genome annotation project (http://rice.plantbiology.msu.edu/). Raw data were processed and mapped on the rice reference sequence using CLC Assembly Cell v. 4.06 with default parameters (http://www.clcbio.com). After data were mapped, normalization was performed and then RPKM (reads per kilobase per million mapped reads) was calculated based on a previous study [15]. To identify differentially expressed genes in RSV-infected vs. mock-treated samples at each time point, fold-changes with respect to RPKM values were calculated. Those genes with a log2-converted fold-change > 1 and < -1 were considered to be differentially expressed genes (DEGs).

### Gene Enrichment Analysis

We divided the DEGs into those that were down-regulated and up-regulated at each time point. To identify enriched GO terms in each group containing lists of DEGs, we used the Gossip package implemented in Blast2GO [16], which uses the Fisher’s Exact Test and corrects for multiple testing. The rice GOSlim assignment file was downloaded from the rice genome annotation project and imported into Blast2GO.

### Quantitative Real-Time RT-PCR

The primers used for real-time PCR were designed using PrimerSelect (DNASTAR, Madison, U.S.A.) following the manual and are listed in S1 Table. Ubiquitin-5 (UBQ5) was used as a reference gene because a previous report indicated that UBQ5 could be used as an internal control for studying gene expression in rice by quantitative real-time PCR. cDNAs of each sample were synthesized by SuperScript III (Invitrogen, Carlsbad, U.S.A.) reverse transcriptase mixture using oligo(dT 20) (Invitrogen) as reverse primers. Real-time PCR was performed with a Bio-Rad CFX384 Real-time PCR system (Bio-Rad, Hercules, U.S.A.) in Bio-Rad iQ SYBR Green Supermix (Bio-Rad) reagents according to manufacturer protocols. Briefly, the real-time PCR amplification reaction for the each gene was performed in a 10-μL volume that included 5 μL of iQ SYBR Green Supermix, 10 ng of cDNA, and 10 pm of forward and reverse primers. The PCR cycling condition consisted of an initial 3 min at 95°C followed by 40 cycles of 30 sec at 95°C and 30 sec at 55°C. Data were analyzed with Bio-Rad CFX Manager software (Version 3.1).

## Results

### RNA-Seq Time-Course Analysis of the Rice Transcriptome in Response to RSV Infection

**Fig 1:**
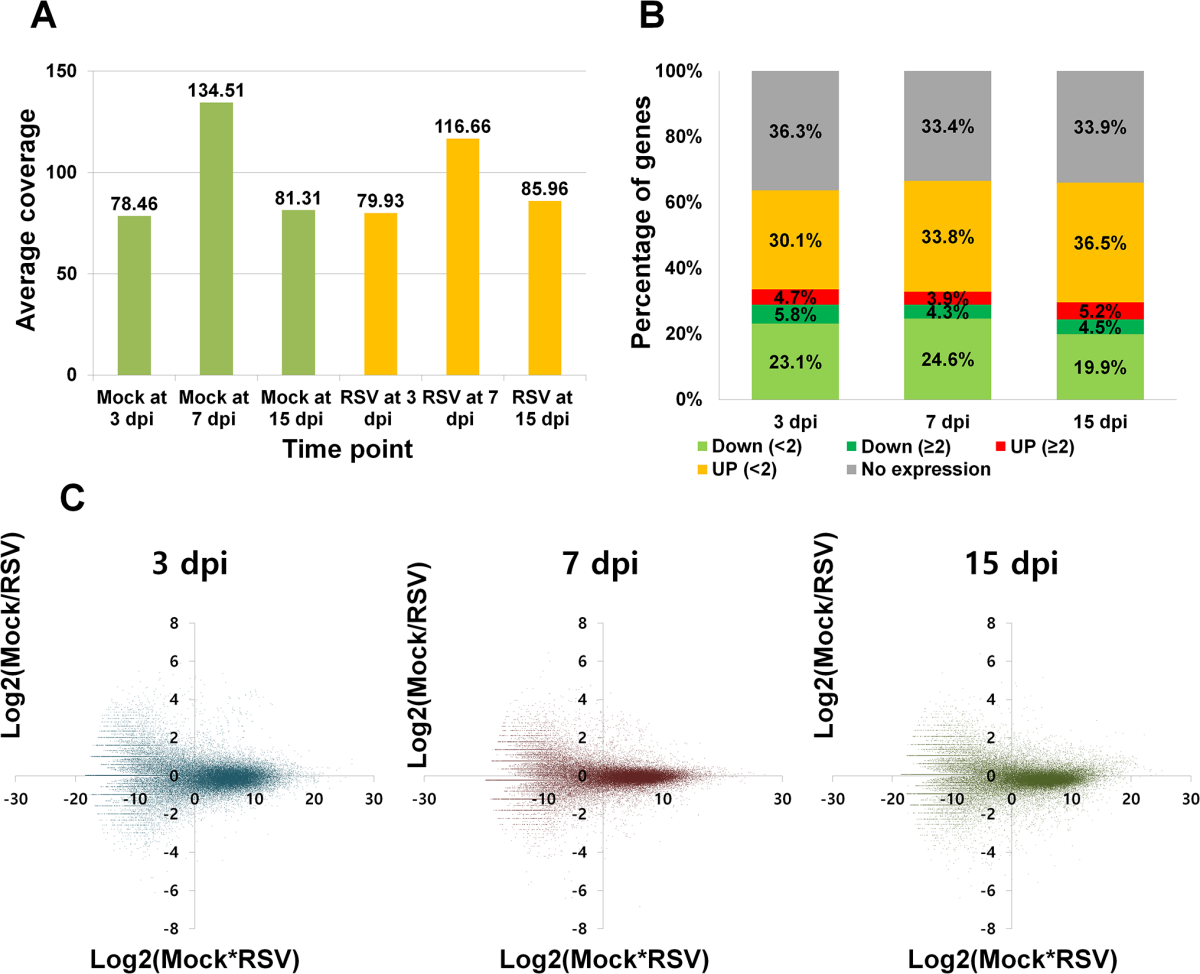
Summary of pair-end sequenced data and the number of expressed rice genes as determined by RNA-Seq for rice plants that were infected with RSV or not infected (mock). Data were collected 3, 7, and 15 days post-infection (dpi). (A) The average coverage of pair-end sequenced RNA-Seq data obtained from each sample. Mock and RSV indicate rice plants that were exposed to non-viruliferous SBPH (green bars) and viruliferous SBPH (orange bars), respectively. (B) The distribution of fold-changes in expression at each time point by comparing RSV to Mock. Genes were divided into five groups: not expressed (gray), down-regulated with < 2-fold change (green); down-regulated with > 2- fold change (dark green); up-regulated with < 2-fold change (orange); and up-regulated with > 2-fold change (red). (C) Ratio versus intensity (R-I) scatter plot of RNA-Seq. X and Y axes represent log_2_-converted Mock*RSV and Mock/RSV values.

To determine the changes in the rice transcriptome associated with RSV infection, we performed a time-course RNA-Seq study using next generation sequencing. Rice seedlings were treated with non-viruliferous SBPH (mock) or viruliferous SBPH containing RSV (RSV), and were harvested at 3, 7, and 15 dpi. To confirm RSV infection in harvested samples, we performed RT-PCR using specific primers for the nucleocapsid gene of RSV (data not shown). A total of six cDNAs (two treatments ×three sample times) were sequenced by Illumina HiSeq 2000 (Illumina). For mapping, we used a total of 66,495 rice genes including 103 plastid-encoded genes and 54 mitochondrial-encoded genes from the Rice Genome Annotation Project. The obtained reads were trimmed and mapped on the reference rice transcripts. From 89.05 to 91.61% of the nucleotides were mapped. (Table 1). Mapping results showed that the nucleotide coverage ranged from 78.46 times (mock at 3 dpi) to 134.51 times (mock at 7 dpi) (Fig 1A). The nucleotide coverage for mock and RSV samples was much higher at 7 dpi than at 3 dpi and 15 dpi. More rice genes were expressed in RSV-infected than in mock plants at 3 and 7 dpi, but fewer genes were expressed in RSV-infected plants at 15 dpi than at 3 and 7 dpi (Table 1).

**Table 1:** Number of trimmed reads, mapped reads, and mapped nucleotides obtained by an RNA-Seq analysis of rice plants that were infected or not infected (mock). Data were collected 3, 7, and 15 days post-infection (dpi).

Index	Mock at 3 dpi	Mock at 7 dpi	Mock at 15 dpi	RSV at 3 dpi	RSV at 7 dpi	RSV at 15 dpi
No. of trimmed reads (%)	102359566 (80.83%)	172656822 (83.24%)	106098542 (81.77%)	100829768 (88.68%)	147455860 (87.97%)	111539460 (87.55%)
No. of mapped reads (%)	91260480 (89.16%)	156513349 (90.65%)	94792088 (89.34%)	92428706 (91.67%)	134964281 (91.53%)	99454637 (89.17%)
No. of mapped nucleotides (%)	8900350573 (89.08%)	15257745450 (90.53%)	9223348646 (89.32%)	9066497550 (91.61%)	13233161957 (91.42%)	9751070103 (89.05%)
Average coverage	78.46	134.51	81.31	79.93	116.66	85.96

### Identification of Differentially Expressed Genes (DEGs) in Response to RSV Infection

Next, we identified DEGs in response to RSV infection by comparing the RSV sample to the mock sample at each time point using a 2-fold change in expression as the cutoff (S2 Table). About 33.4 to 36.3% of rice genes were not expressed, and about 53.2 to 58.4% of rice genes were expressed but the difference in expression was < 2-fold (Fig 1B). The number of DEGs ranged from 5,424 (8.2%) to 6,926 (10.4%) (Fig 1B). To examine global gene expression patterns at each time point, a ratio-intensity plot (RI plot) was generated; the RI plot showed that the global expression patterns were similar among the three time points (Fig 1C). However, the spots were less scattered at 7 dpi than at 3 and 15 dpi.

**Fig 2:**
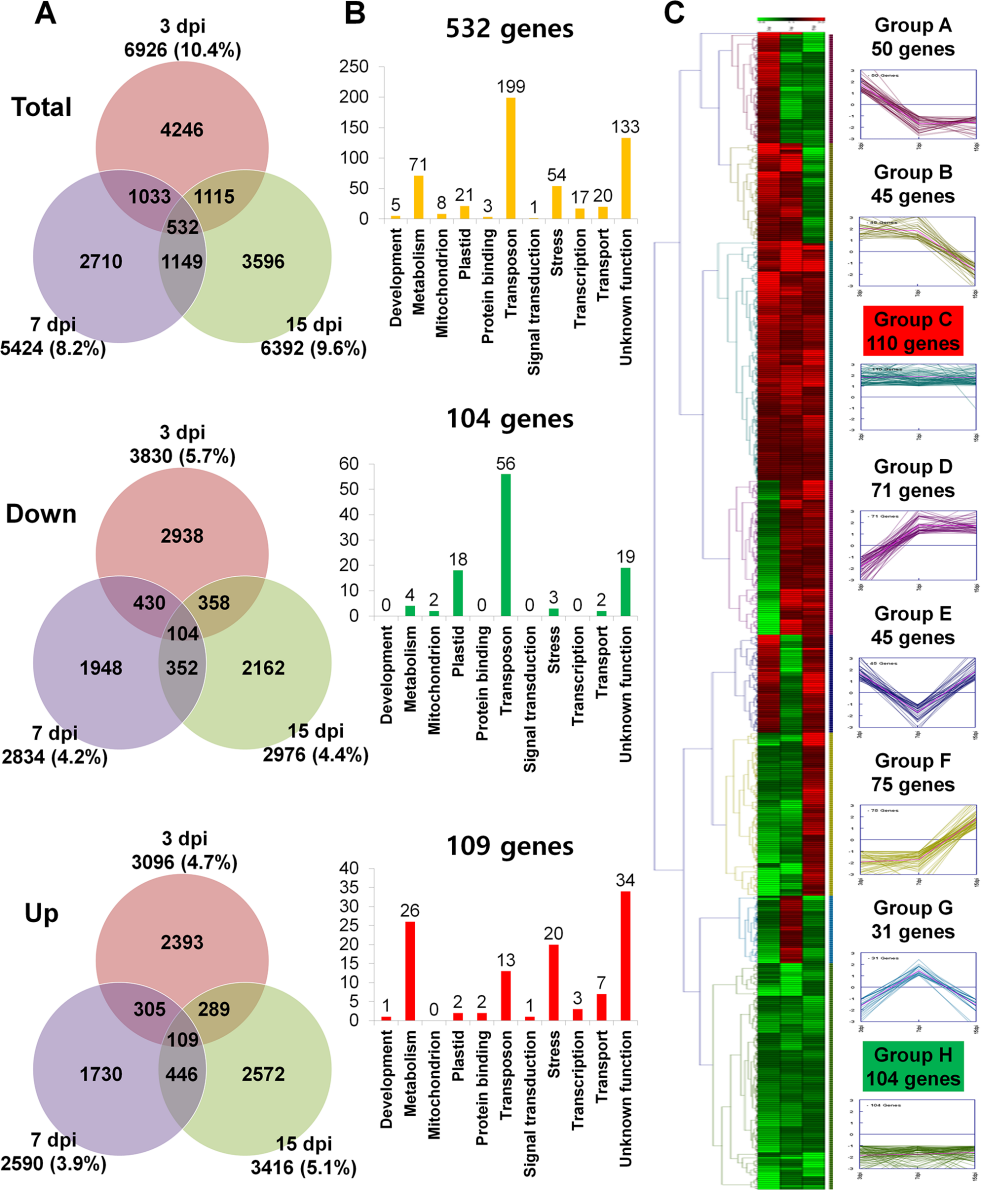
The number of differentially expressed genes (DEGs) in rice in response to RSV infection. Data were collected 3, 7, and 15 days post-infection (dpi). (A) Venn diagram displaying the distribution of DEGs (genes with > 2-fold change in expression) at each time point. Total indicates the total number of both up-regulated and down-regulated DEGs while Down and Up indicate the number of down-regulated and up-regulated DEGs, respectively. (B) The functional classification of DEGs commonly identified at each time point. A total of 532 genes were differentially expressed at all three time points, while 104 and 109 genes were down-regulated and up-regulated, respectively, at all three time points. The DEGs were manually assigned to 11 functional categories. (C) Hierarchical clustering according to changes in expression over time of the 532 genes that were expressed at all three time points. Cluster analysis was performed with the Genesis program [38].

A total of 14,381 genes were differentially expressed at one or more of the three time points. Many DEGs were expressed at only one of the three time points (Fig 2A, top). Among the DEGs that were expressed at only one of the three sampling times, more (4,246 genes) were expressed at 3 dpi than at 7 or 15 dpi (Fig 2A, top). We further divided DEGs into up-regulated and down-regulated groups, and 8,292 genes were only down-regulated (Fig 2A, middle) and 7,844 genes were only up-regulated (Fig 2A, bottom). Of the 14,381 DEGs, 532 were differentially expressed at all three time points. Moreover, 104 genes were down-regulated and 109 genes were up-regulated at all three time points.

### Functional Classification of the 532 Genes That Were Differentially Expressed at All Three Time Points

We further examined the functional roles of the 532 genes that were expressed at all time points based on 11 categories (Fig 2B). Of the 532 genes, transposon-related genes were the most abundant followed by those with unknown function and then those related to metabolism and stress (Fig 2B, top). Of the 104 genes that were down-regulated at all time points, transposon-related genes were the most abundant by genes with unknown function and plastid genes (Fig 2B, middle). Of the 109 genes that were up-regulated at all time points, genes with unknown function were the most abundant followed by genes related to metabolism, stress, and transposons (Fig 2B, bottom). Moreover, a hierarchical clustering identified eight groups of 532 genes according to the changes in their expression over time (Fig 2C). For example, group G contained 31 genes whose expression was repressed at 3 dpi and 15 dpi but highly induced at 7 dpi. Group C (110 genes) contained genes that were mostly down-regulated rather than up-regulated while group H (104 genes) contained genes that were mostly up-regulated rather than down-regulated (Fig 2C).

### Gene Ontology Enrichment Analysis of Identified DEGs at Each Time Point

**Fig 3:**
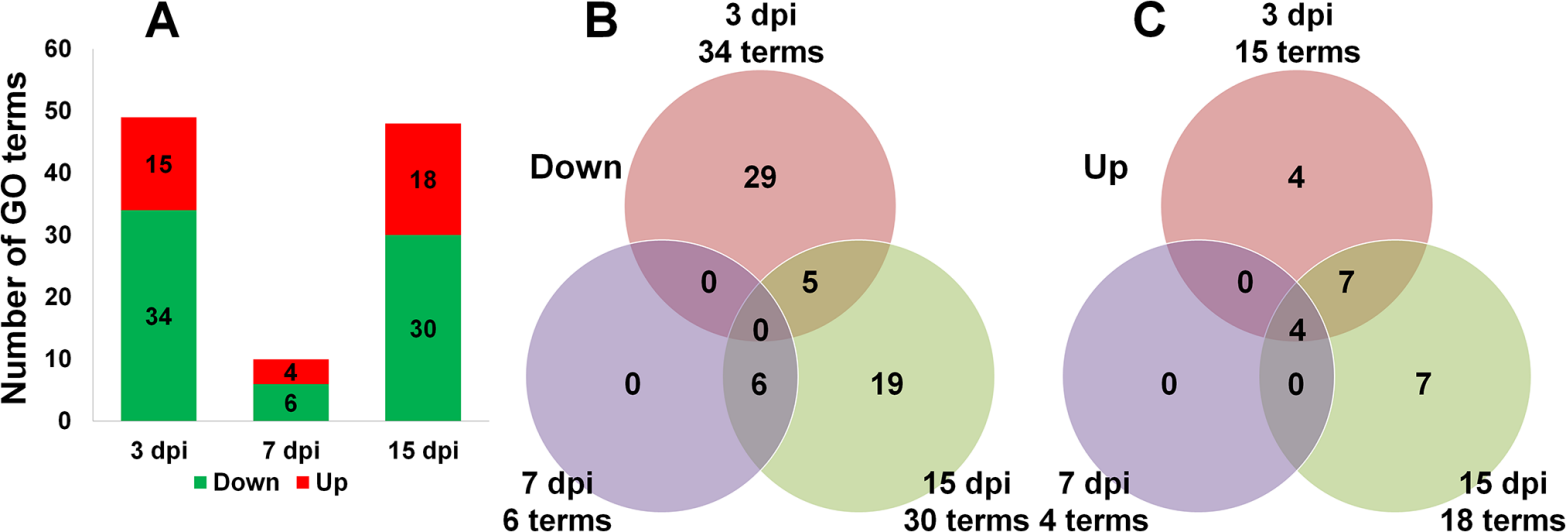
Number of identified GO terms at each time point by GO enrichment analysis. (A) The number of enriched GO terms at each time point. To identify enriched GO terms at each time point, the DEGs at each time point were divided into up-regulated and down-regulated groups. Green and red bars indicate groups of down- and up-regulated genes, respectively. Venn diagrams illustrate the distribution of enriched GO terms for the down-regulated DEGs (B) and up-regulated DEGs (C) at each time point.

To determine the functional roles of DEGs at each time point, we conducted gene ontology (GO) enrichment analysis using the Blast2GO program. The numbers of enriched GO terms at 3 dpi (49 terms) and at 15 dpi (48 terms) were comparable, but the number of enriched GO terms at 7 dpi (10 terms) was relatively low (Fig 3A). The number of enriched GO terms was higher for down-regulated genes (59 terms) than for up-regulated genes (22 terms) (Fig 3B and 3C). Among the down-regulated genes, none of the GO terms was commonly identified at three time points, but five GO terms were common for 3 dpi and 15 dpi and six GO terms were common for 7 dpi and 15 dpi. Interestingly, four GO terms were commonly identified at all three time points.

### Significantly Enriched GO Terms Related to Biological Process

**Fig 4:**
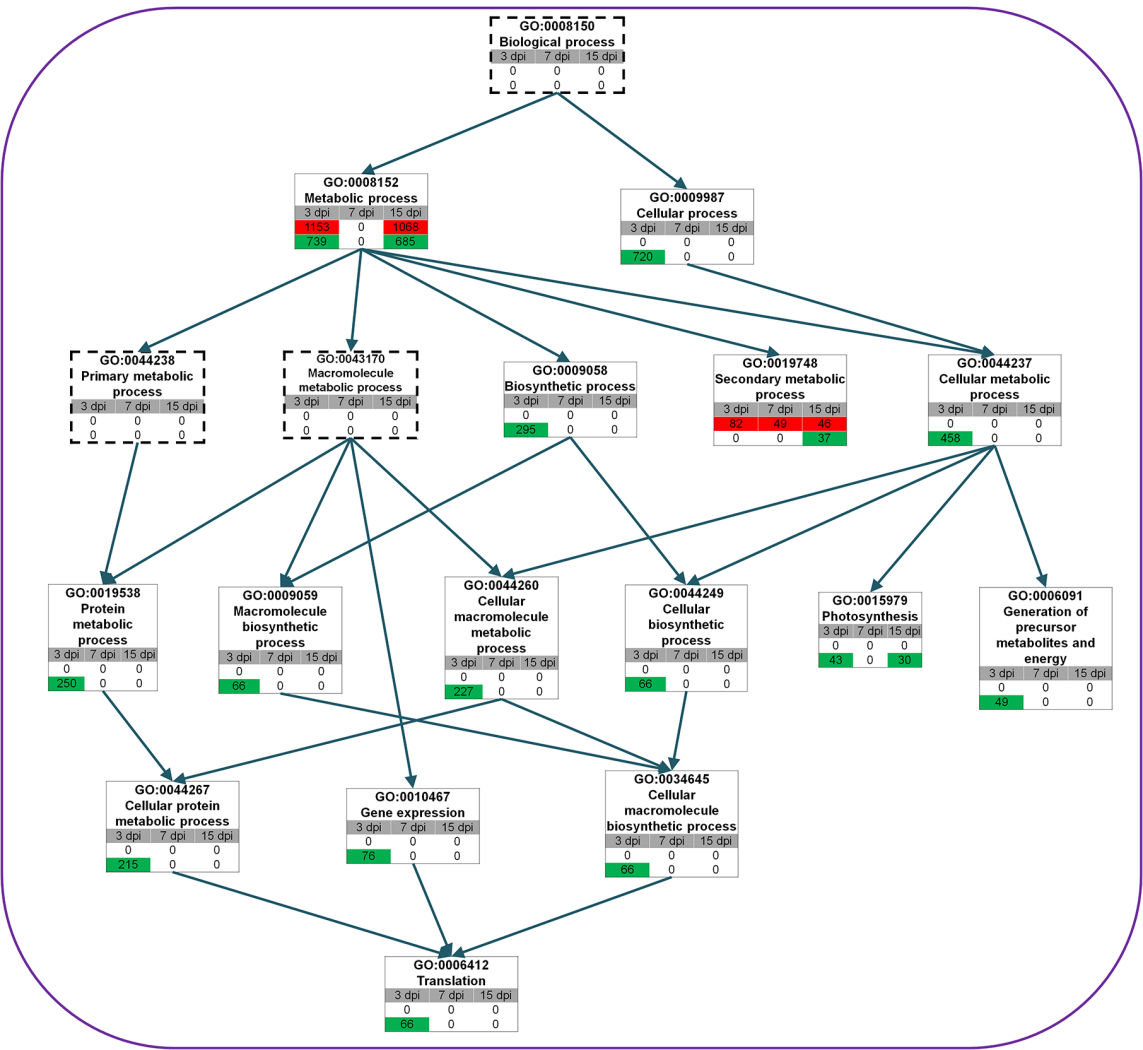
Identified enriched GO terms associated with biological process. GO directed acyclic hierarchical graph (DAG) shows the structural networks of identified GO terms according to biological process. Green and red colors indicate down- and up-regulation, respectively, at each time point, and the number represents the number of DEGs associated with the given GO term. The dashed rectangles represent GO terms that are not significant.

Many down-regulated genes at 3 dpi were related to gene expression (GO:0010467), translation (GO:0006412), and photosynthesis (GO:0015979) (Fig 4). In the case of photosynthesis, 43 genes and 30 genes were down-regulated at 3 dpi and 15 dpi, respectively. Furthermore, GO terms for metabolic process (GO:0008152) and secondary metabolic process (GO:0019748) were overrepresented. In the case of metabolic process at 3 dpi and 15 dpi, 1,153 genes and 1,068 genes, respectively, were up-regulated whereas 739 genes and 685 genes, respectively, were down-regulated. It seems that transcripts for two distinct groups of metabolic genes were differently regulated by RSV infection. The number of up-regulated genes for secondary metabolic process was gradually reduced with increasing infection time. At 15 dpi, GO terms for cell communication (GO:0007154), cell differentiation (GO:0030154), pollination (GO:0009856), and flower development (GO:0009908) were significantly enriched (S1 Fig and S3–S6 Tables). Except for genes related to cell communication, genes belonging to the other three GO terms were strongly down-regulated at 15 dpi. Several stress-associated GO terms such as response to external stimulus (GO:0009605), response to abiotic stimulus (GO:0009628), response to biotic stimulus (GO:0009607), and response to endogenous stimulus (GO:0009719) were enriched both in up- and down-regulated genes (S2 Fig). In the case of the GO term for response to abiotic stimulus, 245 genes were up-regulated at 3 dpi whereas 126 genes and 124 genes were down-regulated at 7 dpi and 15 dpi, respectively. At all three time points, expression of genes required for response to biotic stimulus was strongly affected. For instance, 107 genes were highly up-regulated while 65 genes were down-regulated at 15 dpi. The number of up-regulated genes for various stress responses was always higher than that of down-regulated genes at each time point. In addition, 132 genes associated with transport (GO:0006810) were down-regulated specifically at 15 dpi.

### Significantly Enriched GO Terms Related to Cellular Components

**Fig 5:**
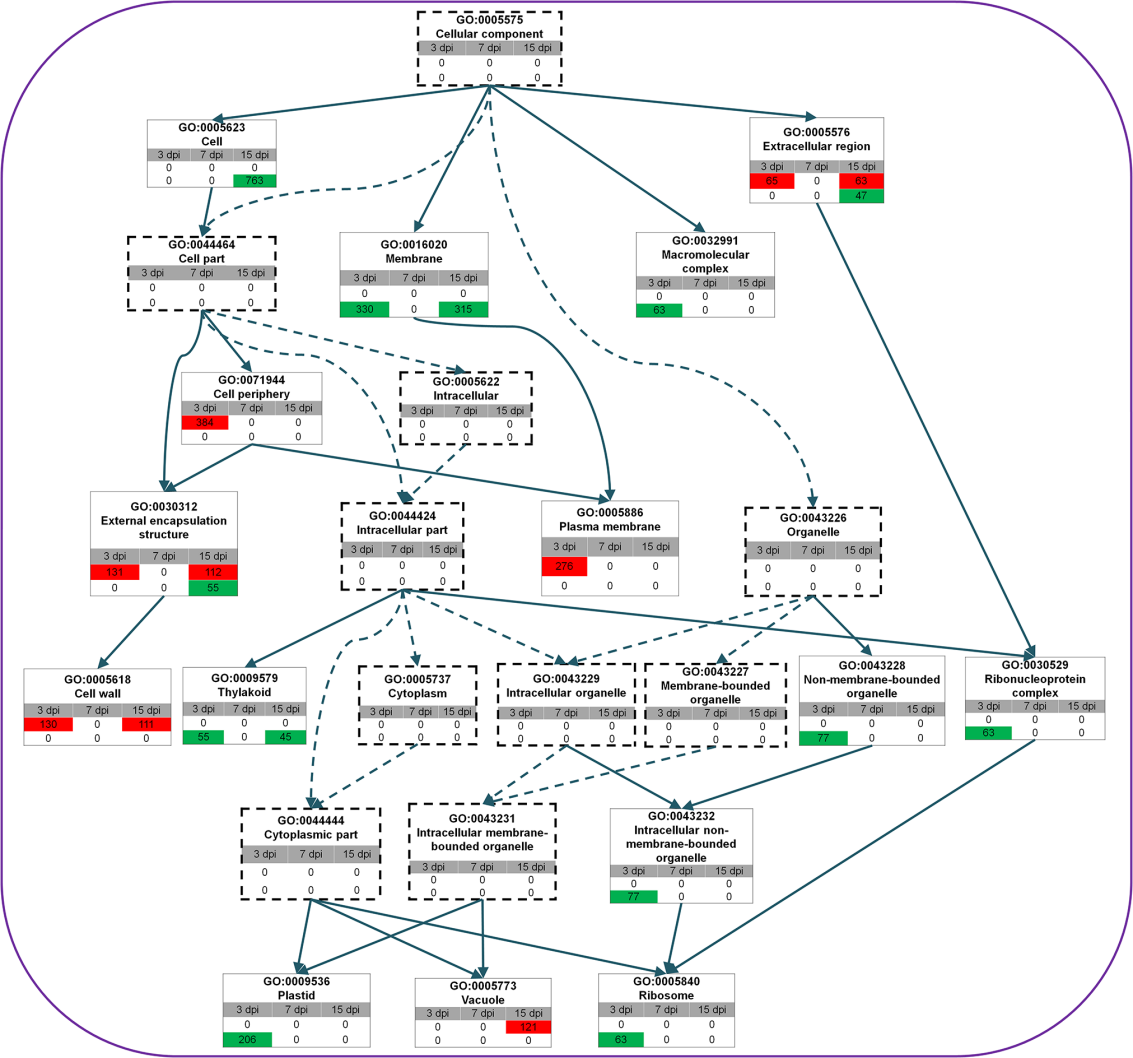
Identified enriched GO terms associated with cellular component. GO directed acyclic hierarchical graph (DAG) shows the structural networks of identified GO terms according to biological process. Green and red indicate down- and up-regulation, respectively, at each time point, and the number represents the number of DEGs associated with the given GO term. The rectangles formed with broken lines represent GO terms that were not significant.

Many up-regulated genes were involved in the cell wall (GO:0005618), plasma membrane (GO:0005886), and vacuole (GO:0005773) (Fig 5). The 276 genes related to the plasma membrane were up-regulated only at 3 dpi, while transcripts of 121 vacuolar genes accumulated only at 15 dpi. Furthermore, transcripts for 130 cell wall-related genes accumulated at 3 dpi and those for 111 cell wall-related genes accumulated at 15 dpi. In contrast, GO terms associated with thylakoids (GO:0009579), ribosomes (GO:0005840), and plastids (GO:0009536) were enriched with down-regulated genes. Plastid- and ribosome-associated genes were specifically up-regulated at 3 dpi while thylakoid-associated genes were down-regulated at 3 dpi and 15 dpi.

### Significantly Enriched GO Terms Related to Molecular Function

GO terms associated with catalytic activity (GO:0003824), transporter activity (GO:0005215), nucleic acid-binding transcription factor activity (GO:0001071), sequence-specific DNA-binding transcription factor activity (GO:0003700), carbohydrate binding (GO:0030246), oxygen binding (GO:0019825), and motor activity (GO:0003774) were overrepresented (S3 Fig). The expression of 111 transcription factor (TF) genes was repressed at 3 dpi while the expression of 173 TF genes was induced at 15 dpi. Genes for catalytic activity were abundant, and their transcripts were preferentially accumulated at each time point. Genes possessing transporter activity were down-regulated at 3 dpi and 15 dpi, but 145 transporter-related genes at 3 dpi were up-regulated. Only a small number of genes associated with carbohydrate binding were identified. Of them, 20 were down-regulated at 3 dpi while 29 were up-regulated at 15 dpi. All oxygen-binding associated genes are members of cytochrome P450. Interestingly, except for the genes that were down-regulated at 3 dpi, many genes related to cytochrome P450 were differentially expressed at three time points. For members of cytochrome P450, the number of down-regulated genes was slightly higher than that of up-regulated genes. Moreover, only 10 genes associated with motor activity were identified as significantly down-regulated at 3 dpi, and they are genes encoding kinesin motor domain-containing proteins.

### Expression of Rice Transcription Factors by RSV Infection

**Fig 6:**
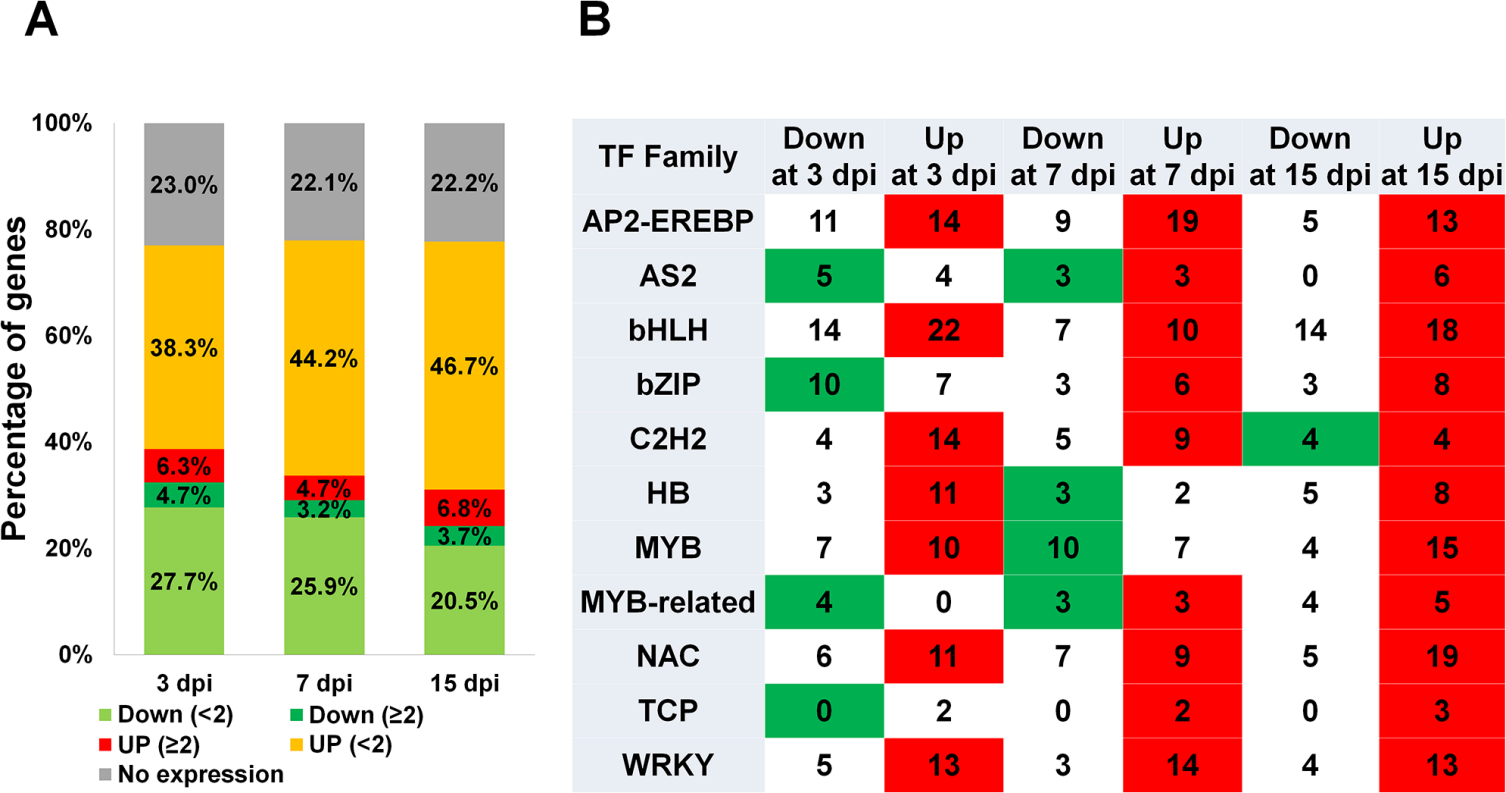
Identification of TFs that were differentially expressed in response to RSV infection. (A) The distribution of fold-changes in expression of TFs at each time point in response to RSV infection. Genes were divided into five groups: not expressed (gray); down-regulated with < 2-fold change (green); down-regulated with > 2-fold change (dark green); up-regulated with < 2-fold change (orange); and up-regulated with > 2-fold change (red). (B) The number of differentially expressed TFs according to the 11 representative TF families at each time point. When the number of differentially up-regulated genes was greater than the number of down-regulated genes, red was used. In the opposite case, green was used.

According to the database of transcription factors (DRTF) (http://drtf.cbi.pku.edu.cn), the rice genome contains 2,383 TFs, which are divided into 63 families (S7 Table). Of the 2,383 TF genes, about 22.1 to 23% were not expressed (Fig 6A). However, 250 to 371 TFs were differentially expressed in response to RSV infection. At all three time points, the number of up-regulated TFs was always higher than that of down-regulated TFs. We further selected 11 representative TF families that showed significant gene expression upon RSV infection (Fig 6B). In general, the number of up-regulated TFs in each TF family was higher than that of down-regulated TFs. This tendency was apparent at 15 dpi, when all 11 TF families except for the C2H2 TF family were strongly up-regulated. Of the 10 strongly up-regulated families, members of AP2-EREBP, bHLH, NAC, and WRKY TF families showed increased levels of transcripts. In down-regulated genes at both 3 dpi and 15 dpi, the number of bHLH TFs (14 TFs) was the highest among other TF families. The members of the MYB TF family were the most abundant of the down-regulated genes at 7 dpi.

### Expression of Rice Transposon Elements, Receptor-Like Kinases, and Resistance Gene Candidates Containing NBS-LRR Domains in Response to RSV Infection

**Fig 7:**
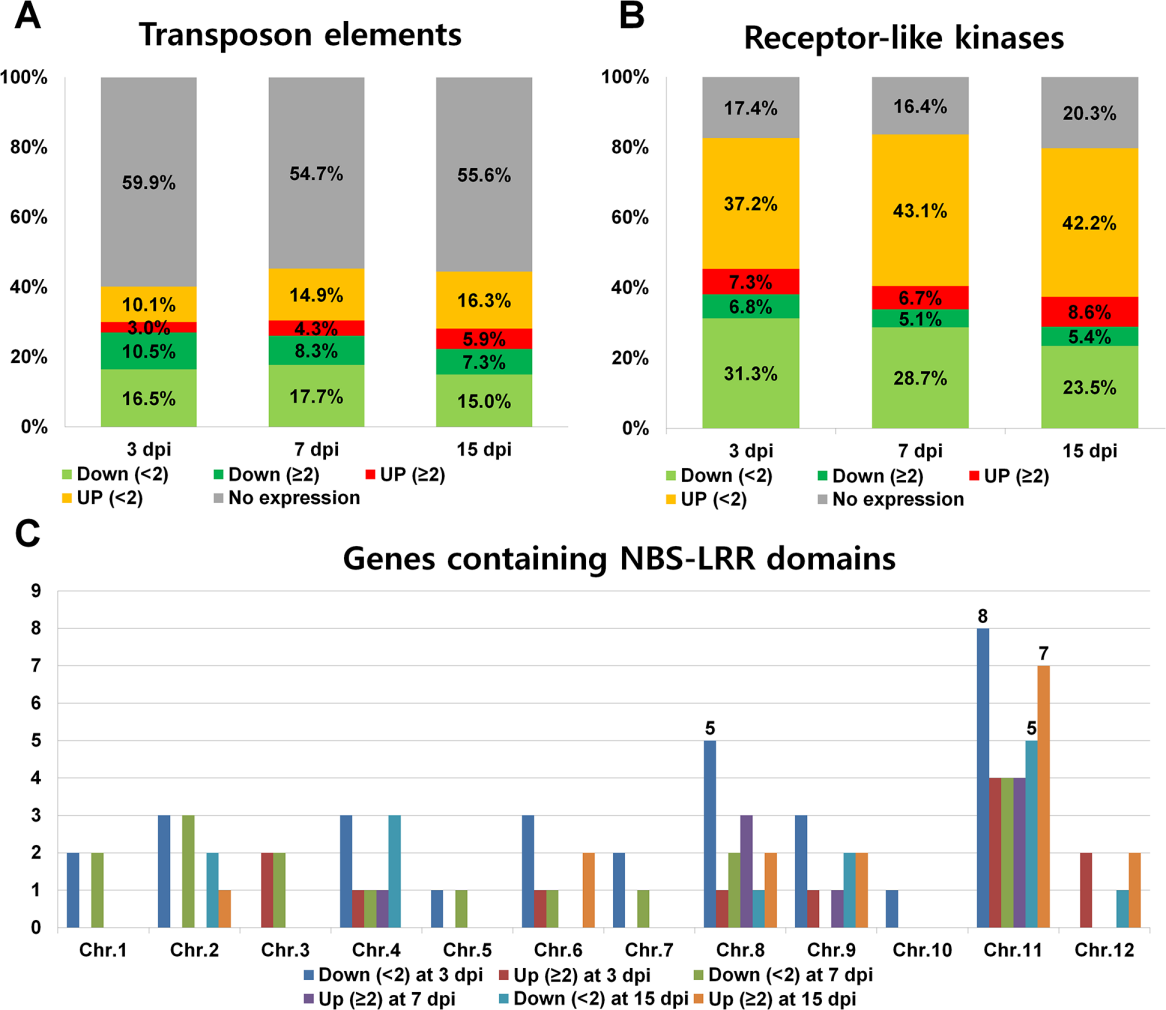
Expression of rice genes associated with transposon elements, receptor-like kinases, and NBS-LRR proteins in response to RSV infection. (A) Expression of genes related to transposon elements (TEs). A total of 17,271 TEs were selected based on the rice genome annotation project. Genes were divided into five groups: not expressed (gray); down-regulated with < 2-fold change (green color); down-regulated with > 2-fold change (dark green color); up-regulated with < 2-fold change (orange color); and up-regulated with > 2-fold change (red color). (B) Expression of genes encoding receptor-like kinases. A total of 1,070 receptor-like kinases belonging to 50 families were selected based on a previous study [39]. (C) The number of expressed NBS-LRR genes on each rice chromosome. A total of 405 rice genes that contained an NBS-LRR domain were selected. The bar graph indicates the number of expressed NBS-LRR genes on each chromosome.

In total, 17,271 genes were related to transposon elements (TEs) in the rice genome. We determined the number of TE-associated genes that were differentially expressed at each time point. Most TEs (54.6 to 59.9%) were not differentially expressed in response to RSV infection (Fig 7A). About 12.7 to 13.5% of TEs showed significant changes in gene expression in response to RSV. The number of down-regulated TEs was much higher than that of up-regulated genes. Amazingly, the number of down-regulated TEs gradually decreased (from 1818 to 1257 genes) while the number of up-regulated TEs increased (from 518 to 1017 genes) with increasing time after RSV inoculation.

In addition, the rice genome contains at least 1070 receptor-like kinases, which were further divided into 50 families. Of them, half of the receptor-like kinase families were differentially expressed, and more than 10% of the receptor-like kinase genes were differentially expressed in response to RSV infection (Fig 7B and S8 Table). About 8.6% of rice receptor-like kinase genes were up-regulated by RSV infection.

At least 480 rice genes possess an NBS-LRR domain, which is highly correlated with resistance against various pathogens [17]. The expression of many genes that contain NBS-LRR domains and that are located on chromosome 8 and 11 was strongly affected by RSV (Fig 7C and S9 Table).

### Expression of Organelle-Encoded Genes in Response to RSV Infection

The expression of genes encoded in plastids and mitochondria was analyzed (S4 Fig). Except in the case of two genes, i.e., *ycf68* (LOC_Osp1g01010.1) and *orf85* (LOC_Osp1g01020.1), the 103 plastid-encoded genes were strongly down-regulated in response to RSV at three time points. The degree of repression in expression of plastid-encoded genes was severe at 3 dpi. Most of the 54 rice mitochondrial genes were strongly down-regulated at 3 dpi. These were genes encoding ribosomal protein S2 and many proteins with unknown function. At 7 dpi, seven mitochondrial-encoded genes were strongly up-regulated. Interestingly, LOC_Osm1g00380.1, a gene with unknown function, was expressed at 3 dpi but not at 7 or 15 dpi.

### Expression of Genes Involved in RNA Interference

Argonautes, dicers, and RNA-dependent RNA polymerases play a critical role in mediating RNA interference. The rice genome contains 11 genes encoding DICER-like proteins, 26 genes encoding Argonaute proteins, and five genes encoding RNA-dependent RNA polymerases (S10 Table). The transcript levels of genes encoding DICER-like and RDR proteins were not significantly changed by RSV infection. However, some genes belonging to the Argonaut protein family showed significant changes in gene expression. For example, the expression of *OsAGO12* was highly suppressed at 3 dpi but was induced at 7 dpi. Transcripts of *OsAGO18* accumulated at both 7 and 15 dpi.

### Expression of Putative RSV Resistance Genes

Several recent studies performed QTL (quantitative trait loci) analyses for RSV [6, 18, 19]. These studies determined the presence of putative resistance genes in specific regions of chromosome 11 of rice. We determined the expression profiles of 37 putative resistance genes on chromosome 11 in response to RSV infection (S11 Table). Of the 37 putative resistance genes, 17 showed differential expression in response to RSV infection. Most were strongly down-regulated, and these include the genes encoding brassinosteroid insensitive 1-associated receptor kinase 1 (LOC_Os11g31530.1 and LOC_Os11g31540.1), a gene encoding retrotransposon protein (LOC_Os11g30960.1), and a gene encoding ATP-binding protein (LOC_Os11g31500.1). In contrast, the putative resistance genes encoding retrotransposon protein (LOC_Os11g31320.1) and armadillo (LOC_Os11g31590.1) were strongly up-regulated at 7 and 15 dpi, respectively.

### Validation of RNA-Seq Data by Quantitative Real-Time RT-PCR

We compared RNA-Seq data with real-time RT-PCR data for 20 representative genes that showed strong up-regulation or down-regulation in response to RSV infection according to RNA-Seq. For real-time RT-PCR, we prepared new mock and RSV samples following the same procedures that were used to prepare samples for RNA-Seq. In most cases, the RT-PCR results were correlated with the RNA-Seq results (S5 Fig). In some cases, however, genes that were upregulated or down-regulated according to one method were down-regulated or up-regulated according to the other method. The lack of correlation for some genes might be explained by differences in the samples (different biological samples were processed for the two methods) or by differences in normalization methods.

### Comparative Analysis between RNA-Seq- and Microarray-Based Studies

In our knowledge, at least two different studies performed genome-wide rice transcriptome analyses in response to RSV infection [8, 9]. The first study used a rice microarray to reveal transcriptional changes at four different time points using rice cultivar Nipponbare, which is susceptible to RSV [8]. The second study used RNA-Seq technique to compare transcriptomes of RSV resistant and susceptible cultivars in response to RSV infection [9]. We compared all DEGs from three studies. The previous RNA-Seq study provided only 221 DEGs commonly regulated in both RSV resistant and susceptible cultivars [9]. All DEGs were assigned to rice loci according to MSU rice genome annotation for comparison. We found that 46 rice genes were commonly identified among three studies (S12 Table and S6 Fig). The 46 genes include many unknown function genes as well as genes encoding trypsin inhibitors, kinases, transporter family proteins, terpene synthases, and lipozygenases. We then compared our RNA-Seq results with those of Satoh *et al*. [8], who used a microarray system. Both studies used rice cultivar Nipponbare as plant materials and sampled at different time points after RSV infection. We examined the number of DEGs from two studies at different time points (Table 2). We found that our RNA-Seq results provide very similar number of DEGs at three different time points; however, the microarray data showed that the number of DEGs was increased as the harvested time point increased. We also identified commonly regulated DEGs in both studies. The commonly regulated DEGs (37 DEGs) at 3 dpi were very low; however, the total commonly regulated DEGs (614 DEGs) at 15 dpi were dramatically increased. Next, we combined the DEGs within each study, resulting in 13,352 DEGs from the RNA-Seq study and 9,308 DEGS from the microarray study (S7 Fig). We found that 2,645 DEGs were commonly regulated from both studies. To find functional differences between two studies, we performed GO enrichment analysis and identified 22 GO terms from RNA-Seq and six GO terms from microarray. Among them, five GO terms including secondary metabolic process (GO:0019748), external encapsulating structure (GO:0030312), cell wall (GO:0005618), catalytic activity (GO:0003824), and cell periphery (GO:0071944), vacuole (GO:0005773) was identified as a very specific GO term for microarray analysis, but detailed GO enrichment analysis in RNA-Seq found that vacuolar genes were also strongly up-regulated. This comparison indicates that, in the case of the response of the rice transcriptome to RSV infection, a greater number of DEGs are detected by RNA-Seq-based transcriptome analysis than by microarray analysis.

**Table 2:** Comparison of DEGs between RNA-Seq and microarray studies. We compared the number of DEGs between RNA-Seq (this study) and microarray [8]. Up, down, and common indicate up-regulated, down-regulated, and commonly identified DEGs, respectively. To identify commonly identified DEGs, DEGs at 3 dpi of RNA-Seq data was compared to those of microarray data at 3 dpi. DEGs at 7 dpi of RNA-Seq data were compared to those of microarray data at 6 dpi and 9 dpi. DEGs at 15 dpi of RNA-Seq data were compared to those of microarray data at 12 dpi.

Time point and up or down-regulation	RNA-Seq	Microarray	Common
3 dpi Up	3096	322	21
3 dpi Down	3830	307	16
6 dpi Up		434	
6 dpi Down		316	
7 dpi Up	2590		73
7 dpi Down	2834		46
9 dpi Up		1065	63
9 dpi Down		638	54
12 dpi Up		4535	
12 dpi Down		4325	
15 dpi Up	3416		315
15 dpi Down	2976		299

## Discussion

In this study, we used RNA-Seq to conduct a transcriptome analysis of rice following RSV infection. RNA-Seq is a low cost method that provides high-throughput sequence data. RNA-Seq had been used to annotate the rice transcriptome at single-nucleotide resolution. In general, an RNA-Seq approach can detect more transcripts and DEGs than a microarray approach. However, the number of DEGs detected could depend on the analysis tools and threshold. Furthermore, RNA-Seq can detect transcription factors and other genes that produce low numbers of transcripts. Interestingly, only a small number of genes (532) were differentially expressed at all three time points following RSV infection in the current study, and we suspect that these genes might be especially important in the response of susceptible rice cultivars to RSV.

### Repression of Chloroplast Gene Expression by RSV Infection

Our study showed that RSV infection strongly suppressed the expression of chloroplast genes at early and late stages of infection. The significantly affected chloroplast genes were encoded not only in the plastid but also in the nucleus and are involved in the electron transport that provides energy for photosynthesis. Moreover, down-regulation of chloroplast genes encoding 30S and 50S ribosomal protein genes indicates that the chloroplast translational machinery was impaired by RSV. A previous study with a microarray system found that RSV infection suppressed the expression of chloroplast genes [8]. Such down-regulation of chloroplast genes has also been demonstrated in other microarray studies of plant responses to diverse viruses [20]. Perhaps the down-regulation of chloroplast genes by various plant viruses is a common response of chloroplasts to biotic stress. For example, a recent study compared expression data from several microarray studies under various biotic stresses and concluded that down-regulation of many photosynthetic genes represents a host defense mechanism [21].

Previous studies have also demonstrated that RSV coat proteins accumulate in the thylakoid membranes of chloroplasts of infected tobacco plants and that such accumulation is correlated with the inhibition of photosynthetic electron transport and a change in photosystem II structure [22]. The disruption of chloroplast function by RSV suggests that some RSV proteins interact with chloroplast proteins. A recent study reported that the rice chloroplast protein PsbP interacts with an RSV disease-specific protein (SP); the study also revealed the involvement of PsbP in the development of RSV symptoms [23]. Additional research is warranted on the interaction between host chloroplast proteins and RSV proteins.

In the current study, the transcripts of many rice genes including chloroplast genes were changed soon after (3 dpi) RSV infection, even though the infected rice seedlings did not show any disease symptoms until 6 dpi. According to a previous study, rice plants infected with RSV display leaf necrosis and stunting at 6 dpi and display leaf death due to loss of chloroplast function at 15 dpi [8]. We suspect that down-regulation of chloroplast genes soon after RSV infection reduces photosynthesis, which subsequently results in chlorosis of infected leaves. Although mitochondrial-encoded genes were also down-regulated at an early time point, some of them were later up-regulated. These results suggest that RSV specifically affected the transcriptome of chloroplasts rather than that of mitochondria.

### Down-Regulation of Transcription Factors Required for Cell Differentiation, Flower Development, and Pollination in Response to RSV Infection

Our transcriptome analysis revealed that genes associated with cell differentiation, flower development, and pollination were strongly down-regulated at a late stage of RSV infection. Many of these genes are TFs, including MADS and MYB TFs. For instance, MADS box TFs are known to be involved in the development of floral organs like the silique, carpel, and ovule [24]. Previous studies showed that AID1, which contains an MYB domain, is involved in anther development in rice (Zhu et al., 2004) and that the rice MYB protein OSMYB5 binds to glutelin gene promoters, which promote the expression of recombinant protein genes in rice endosperm (Suzuki et al., 1998; Xing et al., 2008). The significant down-regulation of many MADS and MYB TFs in the current study might be associated with repression of flowering. In addition, the two genes such as *FTL2* and *BRI1* involved in flower development [25, 26], and many kinases including S-locus-like, serine/threonine, and receptor-like kinases associated with pollination were also differentially expressed in response to RSV infection.

Given their function in rice development, the down-regulation of other TFs such as homeobox, bHLH, LUG, and C2C2-YABBY by RSV infection might explain the dwarf phenotype of RSV-infected plants. For example, the loss of the rice homeobox gene *OSH15* function resulted in dwarf plants because of a defect in the development of internodes [27]. Ectopic expression of rice homeobox gene OSH1 in rice, *Arabidopsis*, and tobacco caused morphological changes correlated with changes in hormone levels [28]. Moreover, bHLH TFs mediate brassinosteroid regulation of cell elongation in rice [29] and root hair development [30]. LUG and C2C2-YABBY TFs are involved in rice leaf development [31, 32].

### Up-Regulation of Cell Wall-Associated Genes and Involvement of TEs in Response to RSV Infection

Up-regulation of many genes involved in metabolic processes at early and late times after infection indicates that they are required for stress response. Many of these genes encode proteins targeted to the cell wall, plasma membrane, and vacuole. The plant cell wall, which is the first cell barrier to protect against external stimuli, contains many proteins such as glycosyl hydrolases, pectinesterases, and peroxidases that are required for stress tolerance. Thus, it seems that transcripts of many genes encoding cell wall proteins accumulated soon after RSV inoculation to defend against RSV infection.

It was surprising that the expression of many transposable element (TE)-related genes was strongly regulated by RSV infection. TEs are important components of plant genomes and have critical roles in genome evolution with increasing complete plant genomes [33]. TEs are mostly inserted in introns and/or in 5′ and 3′ untranslated regions, and only a few TEs are reported to be active. However, external stimuli such as biotic and abiotic stresses can repress the silencing of TEs [34]. A recent study demonstrated that rice transposon-derived small RNAs regulate the host–TE interaction [35]. Thus, the functional roles of TEs associated with virus infection should be investigated.

There are several approaches to identify rice genes involved in RSV resistance. For instance, a recent study identified a RSV resistance gene referred as *STV1* encoding a sulfotransferase, which catalyzes the conversion of salicylic acid to sulphonated SA using map based cloning [36]. Several study have used yeast two-hybrid screening, pull-down assay and bimolecular fluorescence complementation assays to identify rice proteins interacting with RSV viral proteins, which are required for RSV infection. For example, the PsbP protein interacting RSV disease-specific protein induces RSV symptoms [23] while HSP70 protein interacting with RSV RNA-dependent RNA polymerase protein is required for RSV infection [37]. In addition, RNS-Seq based approach in our study is an efficient way to provide several candidate genes involved in RSV resistance and life cycle.

In summary, our time-course transcriptome analysis of the response of rice to RSV infection revealed that RSV infection resulted in the down-regulation of genes associated with photosynthesis and flowering and the up-regulation of many genes related to stress response metabolism. In addition, many TE-associated genes were involved in the response to RSV infection. Finally, our transcriptome analysis has provided a list of rice genes that should be useful for the study of rice–RSV interactions.

## Supporting Information

S1 Fig Identified enriched GO terms associated with cell differentiation, pollination, and flower development. GO directed acyclic hierarchical graph (DAG) shows the structural networks of identified GO terms according to biological process. Green and red indicate down- and up-regulation, respectively, at each time point, and the number represents the number of DEGs associated with the given GO term. The rectangles formed with broken lines represent GO terms that are not significant.
(TIF)

S2 Fig Identified enriched GO terms associated with various stress responses and transport. GO directed acyclic hierarchical graph (DAG) shows the structural networks of identified GO terms according to biological process. Green and red indicate down- and up-regulation, respectively, at each time point, and the number represents the number of DEGs associated with the given GO term. The rectangles formed with broken lines represent GO terms that are not significant.
(TIF)

S3 Fig Identified enriched GO terms associated with molecular function. GO directed acyclic hierarchical graph (DAG) shows the structural networks of identified GO terms according to biological process. Green and red indicate down- and up-regulation, respectively, at each time point, and the number represents the number of DEGs associated with the given GO term. The rectangles formed with broken lines represent GO terms that are not significant.
(TIF)

S4 Fig Expression of plastid- and mitochondrial-encoded rice genes. Expression of 103 plastid-encoded genes and 54 mitochondrial-encoded genes was visualized with heatmaps created with the Genesis program. Green and red indicate down-regulation and up-regulation, respectively.
(TIF)

S5 Fig Validation of RNA-Seq data by quantitative real-time RT-PCR. A total of 20 rice genes were selected that were highly regulated by RSV infection according to RNA-Seq data. For real-time RT-PCR, another set of samples was prepared for the same 20 genes. Real-time PCR was performed at least three times for each gene. For each gene, the upper, middle, and bottom bars indicate expression at 3, 7, and 15 dpi, respectively, according to real-time RT-PCR (left) and RNA-Seq (right). Light red, red, and dark red indicate up-regulated genes at 3, 7, and 15 dpi, respectively. Light green, green, and dark green indicate down-regulated genes at 3, 7, and 15 dpi, respectively.
(TIF)

S6 Fig Comparison of DEGs from three different studies. For comparison, all DEGs were assigned to rice loci according to MSU rice genome annotation. The venn diagram was generated by using Venny program (http://bioinfogp.cnb.csic.es/tools/venny/).
(TIF)

S7 Fig Comparison of microarray and RNA-Seq analyses. GO terms identified by the two analyses are separated into biological process (A), cellular component (B), and molecular function (C). (D) Venn diagram illustrating the GO terms identified by the two analyses. In A–C, blue boxes indicate GO terms specifically identified by RNA-Seq analysis, orange boxes indicate GO terms specifically identified by microarray analysis, and red boxes indicate GO terms commonly identified by both analyses.
(TIF)

S1 Table Primers used for the real-time PCR analysis that was compared with the RNA-Seq analysis. (XLSX)

S2 Table Number of reads, coverage, and expression ratios analyzed by RNA-Seq. (XLSX)

S3 Table The expression ratios of 46 rice genes required for cell communication. (XLSX)

S4 Table The expression ratios of 36 rice genes required for cell differentiation. (XLSX)

S5 Table The expression ratios of 22 rice genes required for pollination. (XLSX)

S6 Table The expression ratios of 35 rice genes required for flower development. (XLSX)

S7 Table The expression ratios of rice transcription factors in response to RSV infection. (XLSX)

S8 Table The expression ratios of rice LRR receptor-like kinases. (XLSX)

S9 Table The expression ratios of 405 genes containing NBS-LRR domains. (XLSX)

S10 Table The expression ratios of genes associated with RNA interference. (XLSX)

S11 Table The expression ratios of putative RSV resistance genes. (XLSX)

S12 Table The 46 rice genes commonly identified by three different transcriptome studies. (XLSX)
