# Supplementary figures and images for: Time-Course RNA-Seq Analysis Reveals Transcriptional Changes in Rice Plants Triggered by Rice stripe virus Infection
Source: PLoS One. 2015 Aug 25;10(8):e0136736. doi: 10.1371/journal.pone.0136736 (PMC4549299; doi:10.1371/journal.pone.0136736)

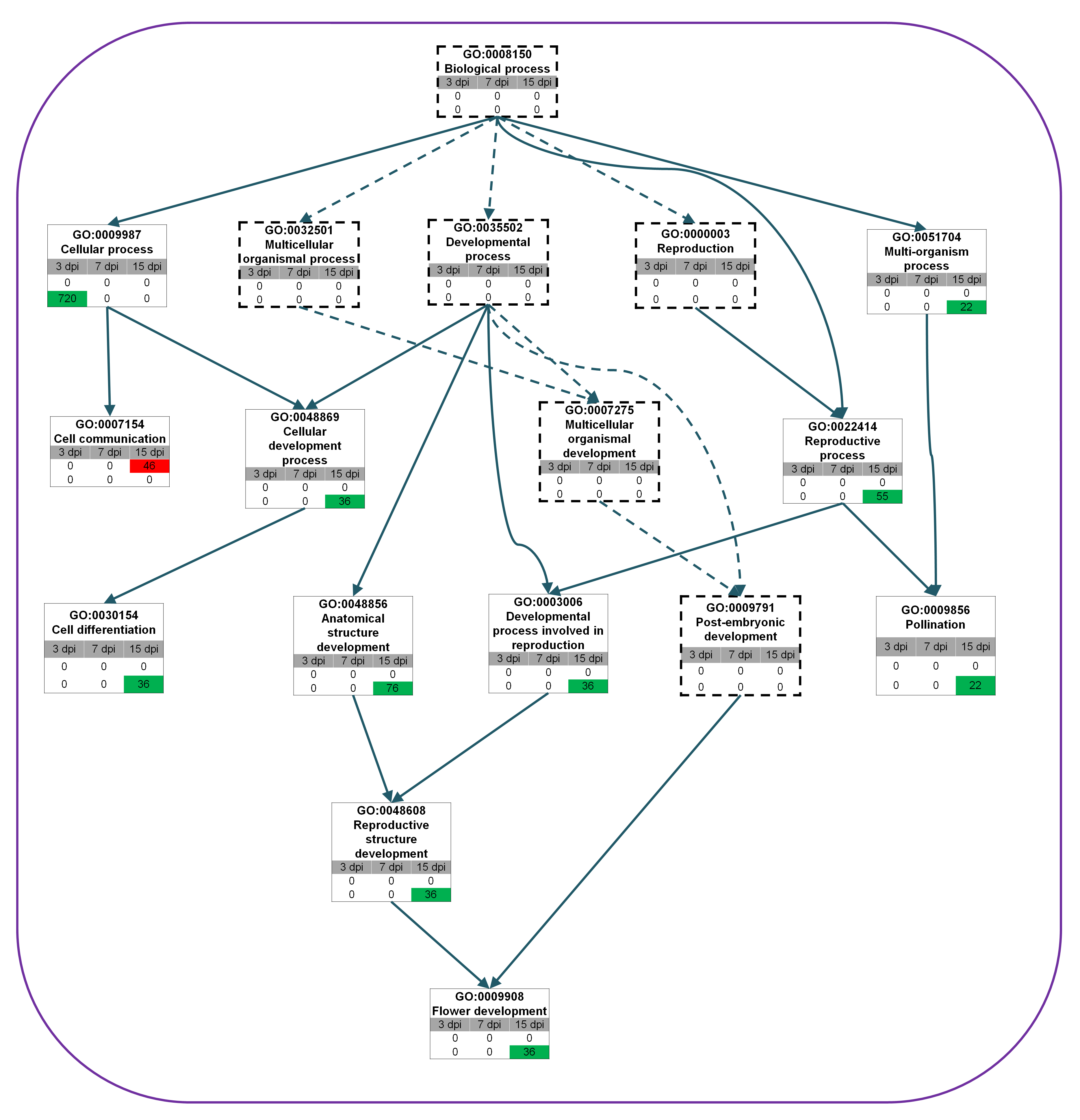

Supplement: S1 Fig — GO directed acyclic hierarchical graph (DAG) shows the structural networks of identified GO terms according to biological process. Green and red indicate down- and up-regulation, respectively, at each time point, and the number represents the number of DEGs associated with the given GO term. The rectangles formed with broken lines represent GO terms that are not significant. (TIF) [file pone.0136736.s001.TIF]

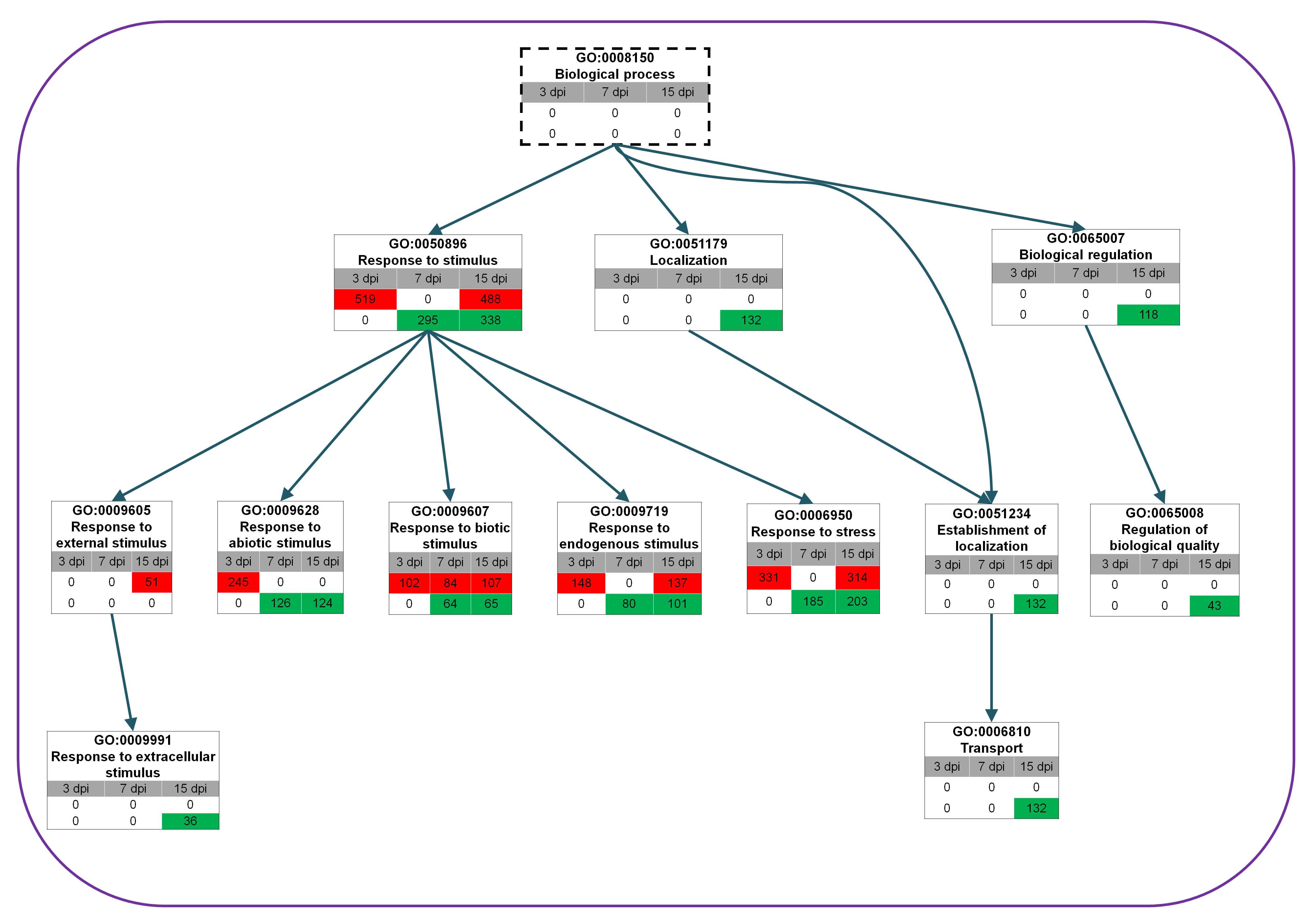

Supplement: S2 Fig — GO directed acyclic hierarchical graph (DAG) shows the structural networks of identified GO terms according to biological process. Green and red indicate down- and up-regulation, respectively, at each time point, and the number represents the number of DEGs associated with the given GO term. The rectangles formed with broken lines represent GO terms that are not significant. (TIF) [file pone.0136736.s002.TIF]

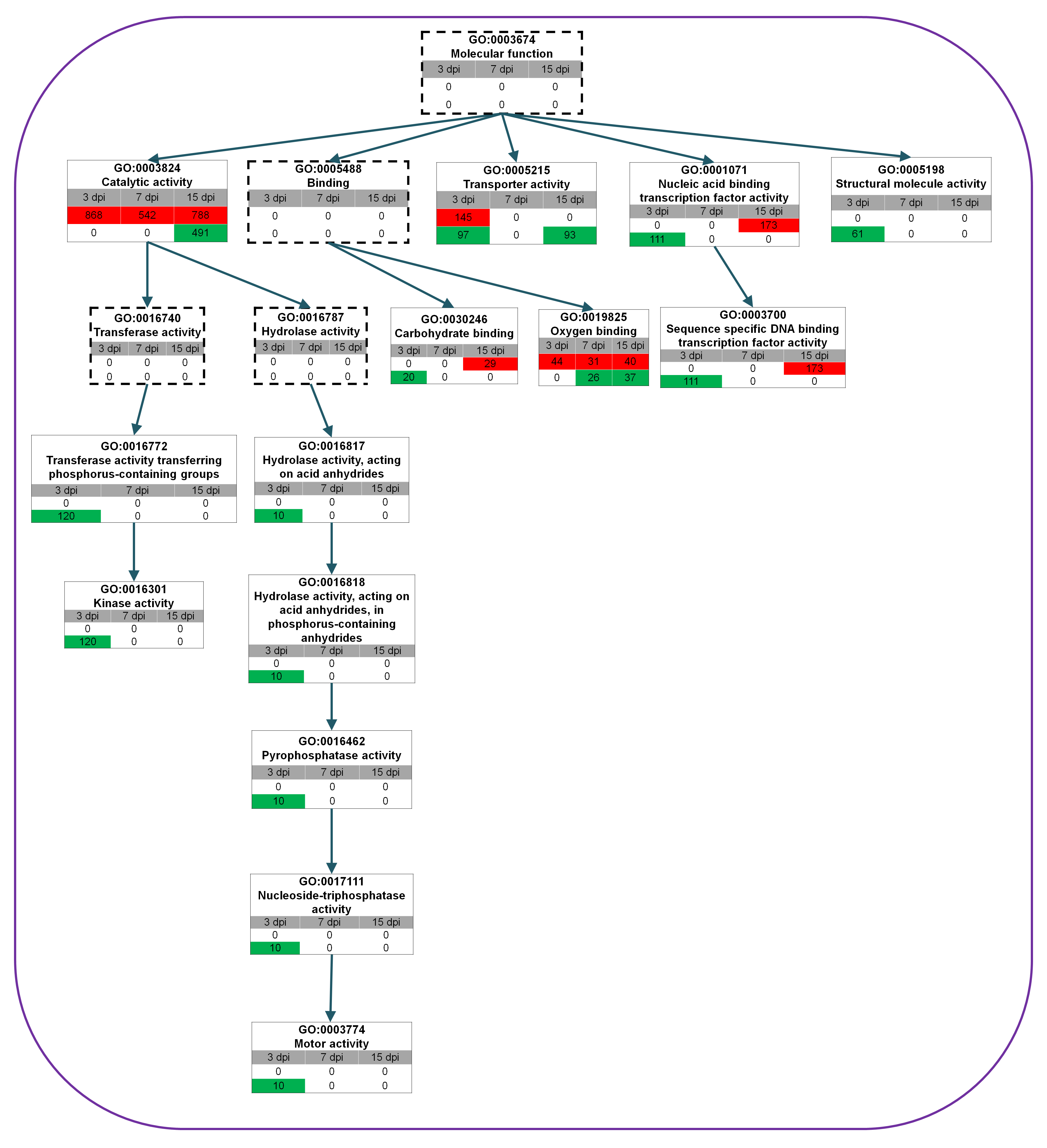

Supplement: S3 Fig — GO directed acyclic hierarchical graph (DAG) shows the structural networks of identified GO terms according to biological process. Green and red indicate down- and up-regulation, respectively, at each time point, and the number represents the number of DEGs associated with the given GO term. The rectangles formed with broken lines represent GO terms that are not significant. (TIF) [file pone.0136736.s003.TIF]

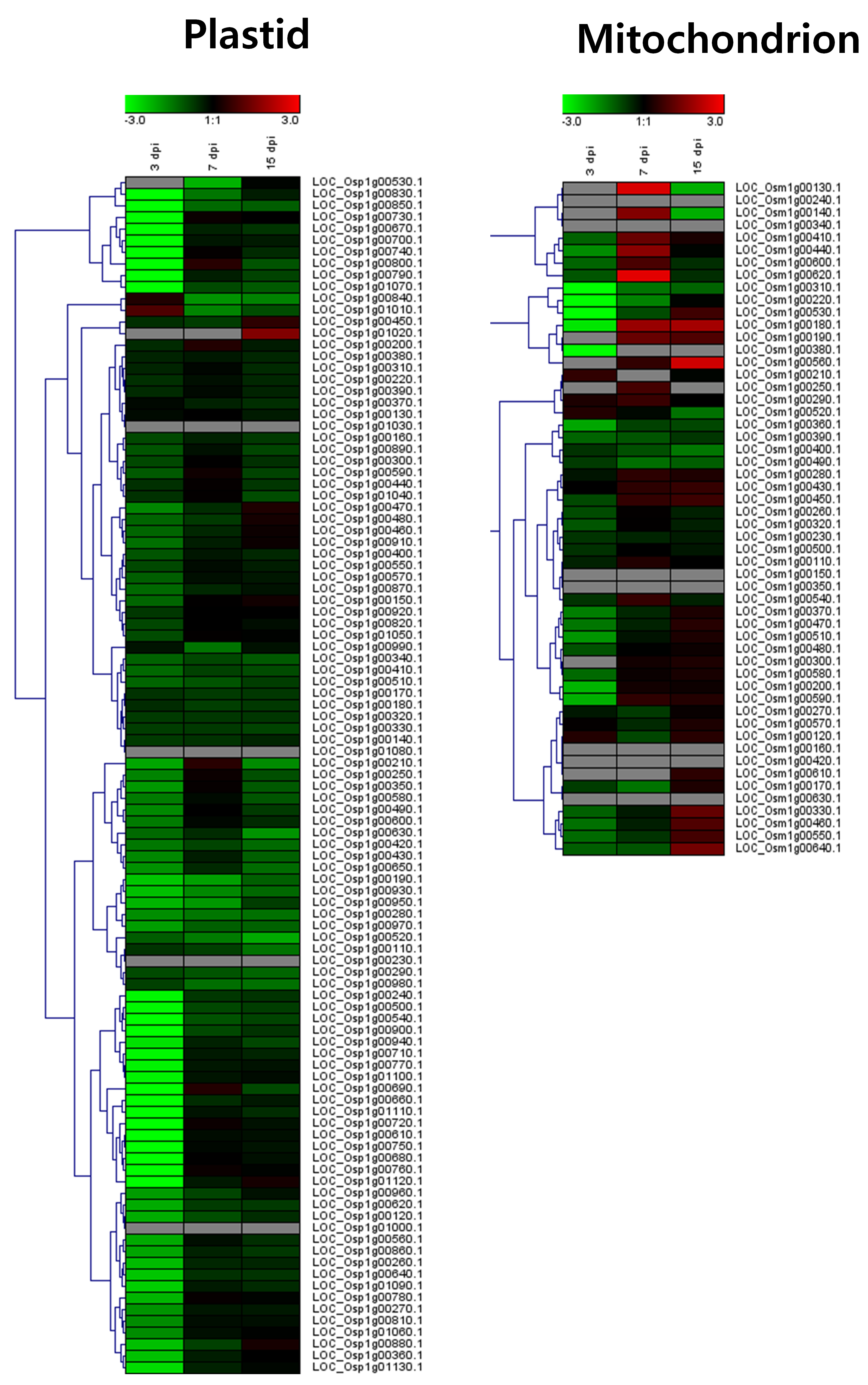

Supplement: S4 Fig — Expression of 103 plastid-encoded genes and 54 mitochondrial-encoded genes was visualized with heatmaps created with the Genesis program. Green and red indicate down-regulation and up-regulation, respectively. (TIF) [file pone.0136736.s004.TIF]

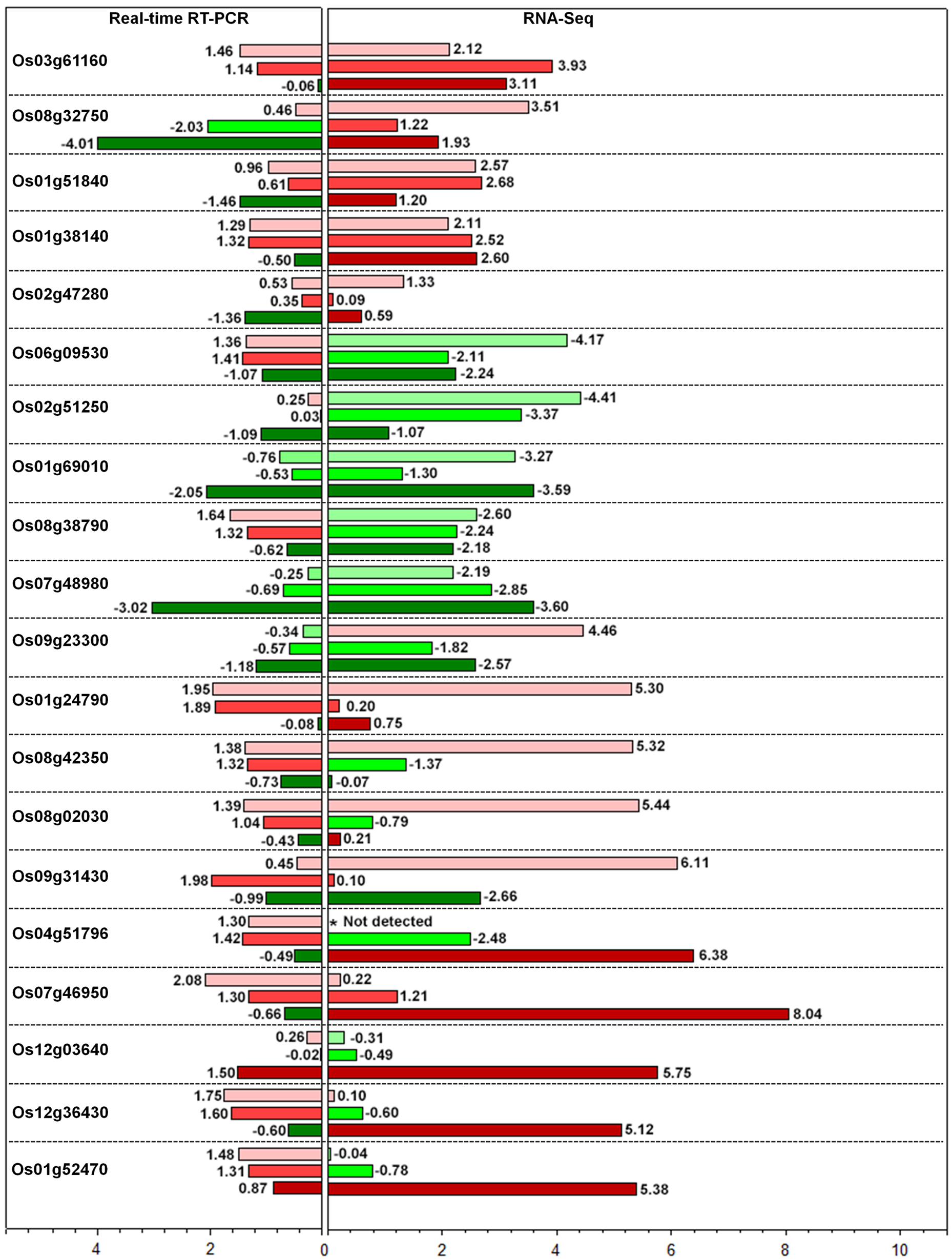

Supplement: S5 Fig — A total of 20 rice genes were selected that were highly regulated by RSV infection according to RNA-Seq data. For real-time RT-PCR, another set of samples was prepared for the same 20 genes. Real-time PCR was performed at least three times for each gene. For each gene, the upper, middle, and bottom bars indicate expression at 3, 7, and 15 dpi, respectively, according to real-time RT-PCR (left) and RNA-Seq (right). Light red, red, and dark red indicate up-regulated genes at 3, 7, and 15 dpi, respectively. Light green, green, and dark green indicate down-regulated genes at 3, 7, and 15 dpi, respectively. (TIF) [file pone.0136736.s005.tif]

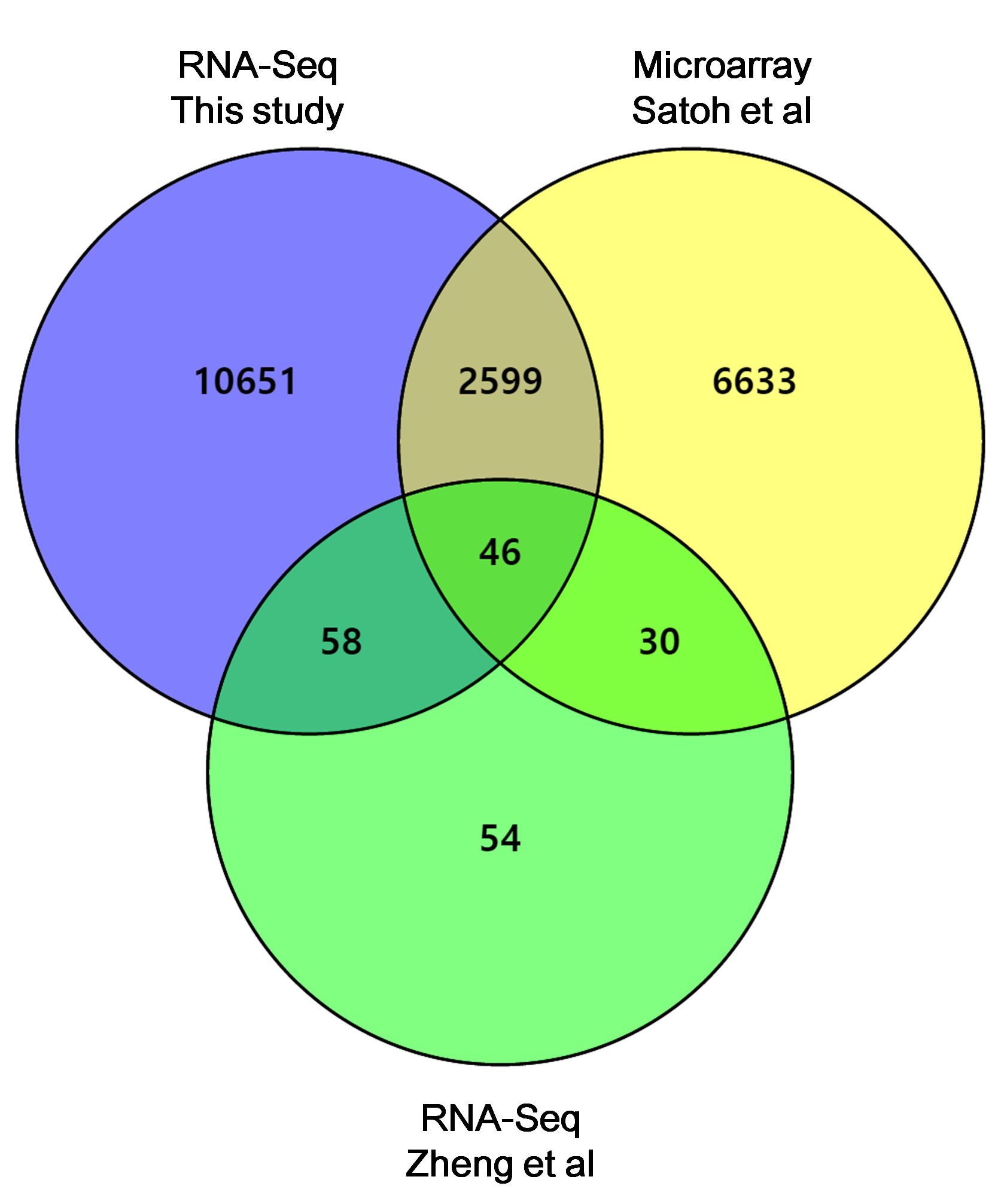

Supplement: S6 Fig — For comparison, all DEGs were assigned to rice loci according to MSU rice genome annotation. The venn diagram was generated by using Venny program (http://bioinfogp.cnb.csic.es/tools/venny/). (TIF) [file pone.0136736.s006.tif]

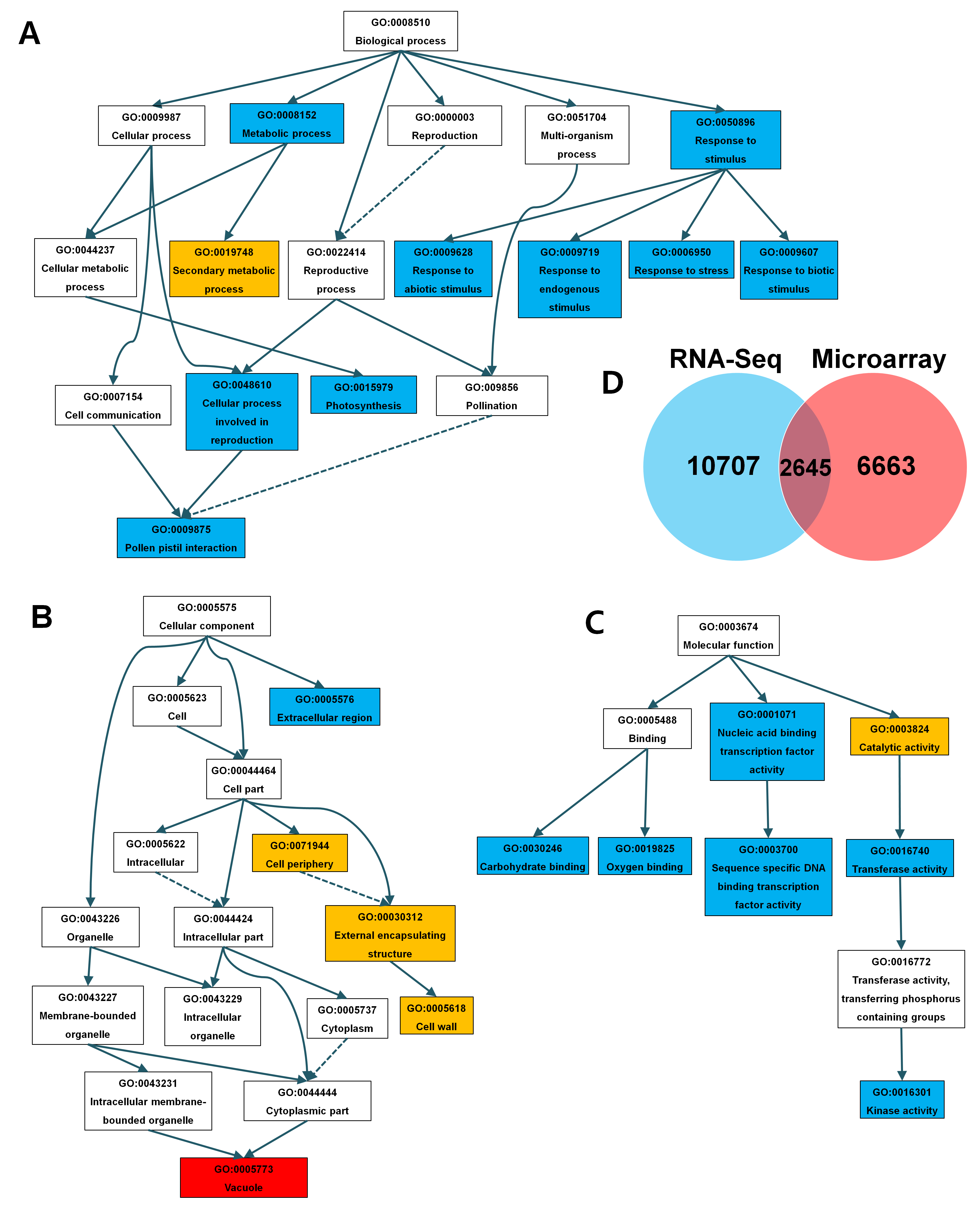

Supplement: S7 Fig — GO terms identified by the two analyses are separated into biological process (A), cellular component (B), and molecular function (C). (D) Venn diagram illustrating the GO terms identified by the two analyses. In A–C, blue boxes indicate GO terms specifically identified by RNA-Seq analysis, orange boxes indicate GO terms specifically identified by microarray analysis, and red boxes indicate GO terms commonly identified by both analyses. (TIF) [file pone.0136736.s007.tif]
